# Being Known: A Grounded Theory Study of the Meaning of Quality Maternity Care to People of Color in Boston

**DOI:** 10.1111/jmwh.13240

**Published:** 2021-07-09

**Authors:** Sanam Roder‐DeWan, Nashira Baril, Candice M. Belanoff, Eugene R. Declercq, Ana Langer

**Affiliations:** ^1^ Neighborhood Birth Center Boston Massachusetts; ^2^ Department of Community Health Sciences Boston University School of Public Health Boston Massachusetts; ^3^ Department of Global Health and Population Harvard T.H. Chan School of Public Health Boston Massachusetts

**Keywords:** birth centers, racism, maternity care, targeted universalism, community‐based participatory research, quality improvement, qualitative research

## Abstract

**Introduction:**

Experiences of people of color with maternity care are understudied but understanding them is important to improving quality and reducing racial disparities in birth outcomes in the United States. This qualitative study explored experiences with maternity care among people of color to describe the meaning of quality maternity care to the cohort and, ultimately, to inform the design of a freestanding birth center in Boston.

**Methods:**

Using a grounded theory design and elements of community‐based participatory research, community activists developing Boston's first freestanding birth center and academics collaborated on this study. Semistructured interviews and focus groups with purposefully sampled people of color were conducted and analyzed using a constant comparative method. Interviewees described their maternity care experiences, ideas about perfect maternity care, and how a freestanding birth center might meet their needs. Open coding, axial coding, and selective coding were used to develop a local theory of what quality care means.

**Results:**

A total of 23 people of color participated in semistructured interviews and focus groups. A core phenomenon arose from the narratives: being known (ie, being seen or heard, or being treated as individuals) during maternity care was an important element of quality care. Contextual factors, including interpersonal and structural racism, power differentials between perinatal care providers and patients, and the bureaucratic nature of hospital‐based maternity care, facilitated negative experiences. People of color did extra work to prevent and mitigate negative experiences, which left them feeling traumatized, regretful, or sad about maternity care. This extra work came in many forms, including cognitive work such as worrying about racism and behavioral changes such as dressing differently to get health care needs met.

**Discussion:**

Being known characterizes quality maternity care among people of color in our sample. Maternity care settings can provide personalized care that helps clients feel known without requiring them to do extra work to achieve this experience.

## INTRODUCTION

According to a 2020 National Academies of Sciences, Engineering, and Medicine report, birth centers, if integrated into wider maternity care systems, are part of the solution to improving poor childbirth experiences and outcomes.^1^ However, without an explicit understanding of the experiences of people of color in pregnancy, labor, birth, and the postpartum period and a commitment to integrating policies and practices to address, redress, and heal, such centers risk maintaining structural inequity and perpetuating disparities. In this study, a group of community organizers working to develop a freestanding birth center—the Neighborhood Birth Center—in Boston, one of the most racially segregated cities in the United States, partnered with researchers to explore the experiences of people of color with maternity care.

Freedom from discrimination in health care is intrinsically valuable as a human right and is associated with various aspects of high‐quality care, including better communication between perinatal care providers and patients, improved adherence to clinical recommendations, and higher satisfaction with care.^2^, ^3^, ^4^, ^5^ Discrimination in health care services based on patient characteristics such as race and ethnicity is also included in the typology of mistreatment of women during childbirth and has been described in studies from around the globe.^6^ In US health care settings, a small but growing body of evidence shows that discrimination, microaggressions (ie, discrimination in everyday interactions often unknowingly perpetrated and sometimes unknowingly received), and implicit bias (ie, bias that is not conscious and thus difficult to control), toward people of color is common.^3^, ^6^, ^7^, ^8^, ^9^, ^10^, ^11^ A nationally representative sample from the Listening to Mothers III survey quantifies the phenomenon in maternity care with 24% of people who have given birth reporting having experienced some form of discrimination.^6^ In this same sample, 10% of Black non‐Hispanic respondents said that they were “always or usually treated poorly in hospital due to race, ethnicity, cultural background, or language”; this rate was 3% in white non‐Hispanic respondents.^1^, ^9^

The physiologic impact of the daily stress caused by racism, termed *allostatic load*, contributes to stark racial disparities in birth outcomes in the United States.^12^, ^13^, ^14^ Maternal mortality among Black people in the Unites States is 2.5 times

**QUICK POINTS**

✦ Maternal mortality among Black people in the United States is 2.5 times higher than white people, and infant mortality among Black infants occurs at more than twice the rate of white infants. Addressing these disparities will require delivering high‐quality technical and interpersonal care, including freedom from discrimination, to all people who are giving birth.
✦ This study uses elements of community‐based participatory research to inform the design of a freestanding birth center in Boston with the voices and experiences of people of color who have given birth.
✦ The study shows that people of color value being known during maternity care (ie, being seen, heard, and treated as individuals). Extra work (ie, steps to prevent or mitigate negative experiences) is done to achieve positive maternity experiences.
✦ Structural and interpersonal racism contribute to not being known. The bureaucratic nature of health care institutions and asymmetry of power and knowledge between perinatal care providers and patients create the context for these negative experiences with maternity care.
✦ In order to provide high‐quality care to all users, maternity care providers should help clients feel known without making them do extra work to ensure a positive experience.

higher than the mortality of white people, and infant mortality among Black infants occurs at more than twice the rate of white infants.^15^, ^16^ These differences persist when controlling for socioeconomic differences and behavior, leading the Centers for Disease Control and Prevention to state that “Identifying and addressing implicit bias and structural racism in health care and community settings … would likely improve patient‐provider interactions, health communication, and health outcomes.”^17^ ^(p 764)^

This study explored the meaning of high‐quality maternity care for people of color in Boston using a grounded theory research design^18^ The approach, which is well suited to a research topic that is yet to be fully addressed in the literature, builds local theory from qualitative data.^18^ Semistructured interviews were conducted with people of color who had given birth; during the interviews, respondents were prompted to share experiences with maternity care, to imagine a “perfect birth,” and to suggest ways that a new birth center could meet their needs. The ultimate goal of this research was to inform the design of Boston's first freestanding birth center so that it can advance equity by delivering high‐quality technical and interpersonal care to all.

## METHODS

This grounded theory study was conducted with the overarching aim of understanding what high‐quality maternity care means to people of color in Boston. Although a full community‐based participatory research approach was not used, elements were incorporated into the research process to ensure that the results were both accurate and useful.^19^ The study was conceived and implemented by community members who are also leaders in the movement to start a freestanding birth center in Boston. A broader group of community members was engaged during the analysis process, and participants were invited to give feedback on the results during a community dissemination event.

The research design, data collection, and analysis were conducted by the first and second authors, both of whom identify as people of color and have lived in the community where the birth center is to be erected, have training in public health, and received maternity care in the city. The first author is a family physician, health systems researcher, and public health practitioner who studies quality of care and is on the board of the Neighborhood Birth Center. The second author has a career focused on racial justice in public health and is the program lead for the birth center project. All authors were influenced by the literature on high‐quality care and on systemic racism in the United States, especially the theoretical work of Camara Phyllis Jones on levels of racism and writings of powell and Menendian on othering and belonging.^5^, ^20^, ^21^, ^22^ The development of this birth center more broadly is guided by the theory of targeted universalism (or progressive universalism in the international literature), which says that designing around the needs of structurally excluded groups leads to service delivery that meets the needs of all and can contribute to a more just society.^23^, ^24^

A purposefully sampled population of people who self‐identified as people of color (ie, did not identify as white) and who had given birth were identified for in‐depth interviews and focus groups. Participants were excluded if they were younger than 18 years. No formal reimbursement was offered for participation. Flyers describing the study and soliciting participation were placed in busy community locations that were frequented by parents, such as entrances to daycare centers and bus stops. Emails to community mailing lists and announcements on community social media groups were also posted. Participants were also asked if they had any friends or family members who might be interested in joining the study (ie, snowball sampling). These interviews focused on 3 clusters of questions that were designed to prompt participants to describe their experiences with prenatal, birth, and postpartum care (ie, maternity care). The first cluster asked respondents to describe their maternity care experiences: “Reflect on examples of things that the doctors/nurses/staff did to show you that they respected you.” The second section asked respondents to imagine and describe a perfect birth: “What would have made your birth ‘perfect?’” The third section asked respondents to share their thoughts on a freestanding birth center: “Do you have any ideas of how a birth center could best serve your needs?”

During the consent process, respondents were told that their participation would help the researchers and organizers develop a freestanding birth center in Boston. All interviews were audio‐recorded and transcribed. Interviewer identity, including race, ethnicity, gender, occupation, and brief birth history, was shared at the beginning of each interview. This study was approved by the Harvard University Institutional Review Board and was determined to be exempt from human subjects research review.

Researchers used a constant comparative method to collect data, document thoughts on what the data meant in the form of memos, and develop meaning (or theory) to influence further data collection. Data were first organized using open codes. This initial categorization was reviewed by an interprofessional team of researchers and community activists. As more content became available, a core phenomenon emerged and became the focus of the iterative analysis process. Using discriminant sampling, interviews continued until this core category was saturated. Axial coding was then used to reorganize the data and build out ideas of what caused the phenomenon and how respondents mitigated the impact of the phenomenon (causal conditions, strategies, conditions and context, and consequences).^18^, ^25^ The final coding step was to selectively code data to connect categories. The model that emerged from this process was again discussed with a group of researchers and community activists, leading to a grounded theory of what high‐quality maternity care means to people of color in Boston. Dedoose was used for coding and analysis (version 7.5.9; SocioCultural Research Consultants, LLC, Los Angeles, CA).

## RESULTS

Semistructured interviews and focus groups were conducted with 23 individuals who identified as people of color (Table 1), more than half of whom identified as Black or African American. Other identifiers used by study participants were Caribbean‐American, Puerto Rican, Dominican, Haitian‐American, Hispanic, and Native‐American. Respondents ranged in age from 25 to 57 years with a mean age of 35 years and described births that occurred at any point during their lives. Respondents included people who gave birth in hospitals or in their homes and people who gave birth vaginally or by cesarean.

**Table 1:** Demographic and Health Characteristics of 23 People of Color Living in Boston Who Have Given Birth

Characteristic	Value
**Age, mean (SD), y**	35 (9)
**Highest level of education completed was high school, n (%)** ^a^	7 (41)
**Self‐identified race or ethnicity, n (%)** ^b^	
Black	7 (30)
Hispanic	2 (9)
Black Latina	1 (4)
German and Black	1 (4)
African American	8 (25)
Native American	1 (4)
Caribbean American	1 (4)
Haitian American	2 (9)
Haitian	1 (4)
Puerto Rican	3 (13)
Cuban	1 (4)
Dominican	1 (4)
**Health characteristics**	
Number of births, mean (SD)	3 (2)
At least one home birth, n (%)	5 (22)
At least one birth by cesarean, n (%)	4 (17)

^a^n = 17 because participants were not required to share demographic characteristics.

^b^Respondents could identify as more than one race or ethnicity, making these percentages total greater than 100%.

**Figure 1:**
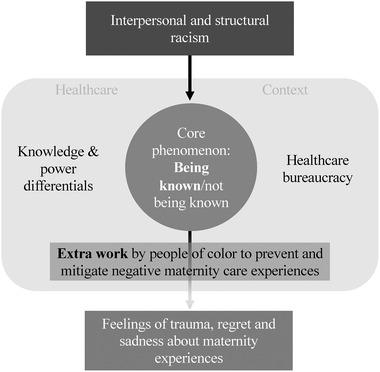
Being Known: A Grounded Theory of the Meaning of Quality Maternity Care to People of Color in Boston

A grounded theory was developed explaining the meaning of quality maternity care among people of color in this sample (Figure 1). Following the tradition of Corbin and Strauss, the grounded theory revolves around a core phenomenon, or central conceptual category^18^ Categories of conditions that describe the context, causes, and consequences of the core phenomenon, as well as the response that respondents take to the phenomenon, complete the theory. In short, the findings were that being known is an important element of quality maternity care to the participants of this study. Being known is threatened by racism (ie, a causal condition) and by health care institutions that are bureaucratic and disempower people by valuing expert technical knowledge over the innate knowledge of people (ie, contextual conditions). In response to these challenges, and as a consequence, people of color did extra work to achieve positive maternity care experiences.

### The Core Phenomenon: Being Known

Respondent interpretations of maternity care quality fell on two sides of a conceptual fulcrum that we call *being known*. Negative maternity care experiences were characterized by not being treated as individuals, not being seen, or not being heard by perinatal care providers. Positive experiences were described as being known by providers. The centrality and importance of being known in these health care narratives made this the core phenomenon. Respondents associated being known with getting what they needed from maternity care encounters and providers. Being invisible, or not being recognized as an individual, meant that mismatches occurred between needs and the services provided.

Yeah, they didn't listen to me. I don't feel like they actually saw me. When I say they didn't see me, I don't think they saw me as a woman, as a first‐time mother, as someone who needed support and needed help.

For the following respondent, not being heard was linked with physical abuse while being positioned for epidural anesthesia:

So, she pushed my head down into the pillow and it was real deep, she was very strong or stiff. I felt like I couldn't move and I wasn't trying to move because I'm like this woman, something is up with her. Her vibe just didn't feel good. And so my face is in the pillow and I can't breathe and then finally she's still holding me while I'm trying to turn my head and she's like, “don't move.” And I said, “I can't breathe” and she's like, “what, what did you say? I can't hear you.

Positive experiences, on the other hand, were associated with being known as an individual. One respondent said, “They complimented me a lot. Umm very playful, very joyful, which you don't see that a lot. It's usually everyone is serious, this is business. They made me feel very comfortable. It was like they knew me.”

The idea of being known also emerged when respondents discussed their thoughts on how a freestanding birth center might meet their needs. Respondents said that a small space that could provide individualized care, where they would be heard, and where perinatal care providers listened would be appealing.

### Racism as a Causal Condition

Respondents often attributed not being known to racism and racial differences between themselves and perinatal care providers or health care staff. These interpersonal dynamics were perceived to mirror dynamics in settings outside of health care. During maternity care, many described interpersonal racism in the form of microaggressions and discrimination that left them feeling disrespected, unseen, and unknown. Respondents told of providers judging them or making assumptions about their backgrounds instead of trying to actually learn about them as individuals.

That's what I felt was taken away from me, like see me for me. I'm a mom who just gave birth to twins, who are premature and I'm their mommy. Not like this Black lady who is in here who is super pissed off and her hair is a wreck.

Respondents felt that they were treated as “second class citizens” and did not receive the care they needed because they were seen as stereotypes instead of as individuals:

I was already perceived as the stereotypical black woman because I didn't have a husband or a male there, so I think there was already the assumption about who I was as a mother or what kind of mother I would be … and even when it came to pain medication and things like that, it was so hard for me to get anything.

Many described loneliness or alienation from perinatal care providers and the maternity care system because of race. They did not see doctors or nurses of color and frequently found themselves with peer groups that did not share their life experiences as people of color. Respondents expressed relief, comfort, and the ability to get needs met when they interacted with other people of color in the system. In addition to these experiences of interpersonal racism, respondents situated negative maternity care within a broader paradigm of structural racism. One respondent said:

I just felt like there was a condescending tone with how they were talking to me … but I always assume that there were a number of things that they were relying on when they saw me walk into a room. Their knowledge is based on their training and that training is in the United States of America and that training is not untainted.

### Contextual Conditions Related to Health Care Institutions

Two specific characteristics of the institution of health care facilitated experiences of not being known. First, interviewees described needing to suppress individual or unique desires because of the policies, agendas, and rules of the health care institution. If there was a conflict between the respondent and the perinatal care provider, providers were perceived to use these rules to coerce patients. Second, respondents frequently described feeling powerless vis‐à‐vis providers and often related this powerlessness with having less knowledge or expertise than the provider.

Once you go into the hospital you think everyone knows what they are doing. The nurse knows, the doctor knows and you give your power to them … you are just a patient that came in and you just fall in line with everything instead of your birth being unique to you.

Perinatal care providers were perceived to use their greater technical knowledge and expert position to maintain power and to exclude, control, or coerce the respondent. When asked how a birth center might deliver high‐quality care, many respondents highlighted the need for more flexibility, patient education, and information sharing.

### Responding to Not Being Known with Extra Work

Informants employed many different strategies to prevent or mitigate not being known. These strategies were the extra work that people of color did to increase the chances of having a positive maternity care experience. Extra work came in many forms, including arming themselves with knowledge about maternity care. One respondent said:

The language that they were using with me was, oh, you're African American, so you fit in this category—meaning some medication. And so, my answers were always like, I'll research that and get back to you. I would never just do it.

Respondents wrote birth plans, brought advocates with them to health care encounters, shared educational credentials with perinatal care providers, or chose to speak English to accommodate providers. Some left the formal system and gave birth at home. Respondents described needing to do extra cognitive work in the form of worrying that they were being seen as a racial stereotype instead of as an individual or giving providers the benefit of the doubt when they felt that they were being discriminated against. Several informants discussed changing their appearance or how they spoke to receive the treatment that they desired.

Now that I'm thinking about it I started dressing up to go to the NICU, almost business casual, so I looked more approachable, less intimidating … maybe if I clean myself up a little bit I won't get treated badly … I think she was intimidated because I was Black. I wasn't screaming or yelling, I was using, you know, proper pronouns, proper verbs and proper grammar and still didn't get anywhere.

### Consequences of Not Being Known and Extra Work

The negative maternity experiences described above and the extra work required to respond to them and mitigate further negative experiences left respondents feeling regret and sadness about their maternity care experiences. Multiple respondents wished they had known more about how to advocate for themselves or how to maintain power in maternity care encounters. The word “traumatic” was used repeatedly to describe birth experiences, and many described a disconnect between what had been hoped for and what was experienced. One interviewee said, “I just wish it was like the total opposite of everything that I experienced.”

## DISCUSSION

The results of this study show that, for people of color, quality maternity care includes being known by perinatal care providers. Structural and interpersonal racism are closely linked to not being known, and health care bureaucracy and large power differentials between patients and providers create the context for negative experiences. The negative maternity care experiences described by the participants of this study include several elements of the typology of the mistreatment of women during childbirth, including stigma and discrimination, physical abuse, and failure to meet professional standards of care.^5^ People of color do extra work to increase the chances of having a positive experience, but many respondents describe their maternity experiences with sadness and regret.

Being known by health care providers has been shown to be important to patients in previous studies.^26^ In maternity care, the phenomenon is expressed in a variety of forms, including in studies that show the importance of relationship‐building between providers and patients and in continuity of prenatal and childbirth care.^27^, ^28^ Our findings are also consistent with the literature on autonomy and decision‐making in childbirth.^29^ In the global literature on disrespect and abuse in maternity care, asymmetries in power between people giving birth and providers during pregnancy and birth services are thought to enable poor treatment.^30^ People in our sample recognized that power is controlled, at least partially, by the control of knowledge; the system values provider technical knowledge over the innate knowledge of people giving birth. This finding is strongly supported by another recent qualitative study of 22 women of color in the United States; providers “packaged” information in a way that made it hard for respondents to participate, engage, and have power during maternity care experiences.^31^

The bureaucratic nature of health care created a context for poor treatment. Institutions are rule‐bound and structured to be efficient and effective in delivering services. However, by emphasizing effectiveness and efficiency they can exclude nonexperts, fail to meet varying client needs, and make arbitrary decisions that alienate individuals and cause suffering.^32^ For people of color, the institutional power asymmetry is compounded by race‐based power differentials that adds an additional layer of stress to their experiences with health care.^12^, ^33^, ^34^ In order to mitigate or prevent these experiences, respondents describe taking action that we call *extra work* to prevent poor treatment. This extra work is described in the literature on *stereotype threat*, that is, the threat of fulfilling the characteristics of a stereotype or being judged based on a stereotype.^35^ Initially studied as it applies to academic achievement, stereotype threat is increasingly being recognized as a variable that affects experiences in health care.^36^ Finally, the consequences of negative birth experiences in our study population included feeling sadness and regret about the experience. Many informants described their experiences with maternity care as “traumatic.” Psychological trauma related to childbirth is well documented in the literature on childbirth experiences in the United States.^37^, ^38^, ^39^

These results provide an unsettling echo to previous studies describing poor maternity care experiences among people of color in the United States.^13^, ^31^, ^40^ Vedam et al found that 17% of a sample of women who gave birth between 2010 and 2016 in the United States (n = 2138) experienced mistreatment during childbirth with significant differences by sociodemographic characteristics, including race. Of Black women, 22.5% reported mistreatment, whereas 14.1% of white women did; Black women had a 1.77 times higher odds (95% CI, 1.31‐2.40) of reporting mistreatment than white women.^11^ Similarly, in an analysis from the Listening to Mother survey of a nationally representative sample, Attanasio and Kozhimannil found that Black non‐Hispanic respondents had a 2.99 times higher odds (95% CI, 1.56‐5.74) of reporting poor treatment during a childbirth hospitalization due to race, language, or culture than white women.^6^ Given that people of color who seek maternity care in the United States have experienced a lifetime of disadvantage across multiple social and economic systems and then are likely to give birth in a health care facility that was not historically designed with or for them, these results are not entirely surprising.^10^, ^41^

### Limitations

This study was limited by its lack of generalizability. A commitment to creating a freestanding birth center may bias researchers toward this particular intervention.^42^ The study results are also potentially affected by recall bias; informants were asked to remember details of their birth experiences that may have happened many years prior to the interview. Conversely, the semistructured interviews and focus groups allowed for deep exploration of the experiences of an understudied population, and the grounded theory approach led to a theory of what high‐quality care means to this population. Community‐based participatory research helped the team formulate relevant research questions and interpret, or ground‐truth, results from multiple perspectives.

### Implications

Several important service delivery and research priorities arise from this work. Providing opportunities for racial concordance between perinatal care providers and clients may be considered to facilitate relationship‐building during clinical encounters and allow people to feel known.^43^ Further provider training may be needed, especially at the preservice level, on the delivery of person‐centered care that is free from discrimination and values the preferences and innate knowledge of people who are giving birth. More research is needed to understand care practices that help birthing people to be known and to shift extra work from them to maternity care facilities and systems. An understanding of how to quantitatively measure being known and extra work will facilitate the use of these findings to monitor and improve quality of care in maternity care settings.

## CONCLUSION

The findings of this study show that being known during maternity care is a critical element of high‐quality care for this study population. Although the study was conducted to inform a specific type of maternity setting (a freestanding birth center) and a particular facility (the Neighborhood Birth Center), the results are supported by the literature on women's experiences in maternity care and may apply more broadly. Maternity care providers in a wide variety of health care settings can explore care practices that help people to be known and monitor for extra work that people do to safeguard positive experiences.

## CONFLICT OF INTEREST

The authors have no conflicts of interest to disclose.

## References

[1] National Academies of Sciences, Engineering, and Medicine; Health and Medicine Division; Division of Behavioral and Social Sciences and Education; Board on Children, Youth, and Families; Committee on Assessing Health Outcomes by Birth Settings ; Backes EP, Scrimshaw SC, eds. Birth Settings in America: Outcomes, Quality, Access, and Choice. Washington, DC: National Academies Press; 2020. 32049472

[2] Hausmann MLR, Hannon JM, Kresevic MD, Hanusa HB, Kwoh KC, Ibrahim AS. Impact of perceived discrimination in healthcare on patient‐provider communication. Med Care. 2011;49(7):626‐633. 10.1097/MLR.0b013e318215d93c 21478769 PMC3117903

[3] Hall WJ, Chapman MV, Lee KM, et al. Implicit racial/ethnic bias among health care professionals and its influence on health care outcomes: a systematic review. Am J Public Health. 2015;105(12):e60‐e76. 10.2105/AJPH.2015.302903 PMC4638275 26469668

[4] Attanasio L, Kozhimannil KB. Health care engagement and follow‐up after perceived discrimination in maternity care. Med Care. 2017;55(9):830‐833. 10.1097/MLR.0000000000000773 28692572

[5] Bohren MA, Vogel JP, Hunter EC, et al. The mistreatment of women during childbirth in health facilities globally: a mixed‐methods systematic review. PLoS Med. 2015;12(6):e1001847. 26126110 10.1371/journal.pmed.1001847 PMC4488322

[6] Attanasio L, Kozhimannil KB. Patient‐reported communication quality and perceived discrimination in maternity care. Med Care. 2015;53(10):863‐871. 10.1097/MLR.0000000000000411 26340663 PMC4570858

[7] Esposito NW. Marginalized women's comparisons of their hospital and freestanding birth center experiences: a contrast of inner‐city birthing systems. Health Care Women Int. 1999;20(2):111‐126. 10409982 10.1080/073993399245827

[8] Center for Reproductive Rights; National Latina Institute for Reproductive Health; SisterSong Women of Color Reproductive Justice Collective . Reproductive Injustice: Racial and Gender Discrimination in U.S. Health Care. A Shadow Report for the UN Committee on the Elimination of Racial Discrimination. New York: Center for Reproductive Rights; 2014. http://tbinternet.ohchr.org/Treaties/CERD/Shared%20Documents/USA/INT_CERD_NGO_USA_17560_E.pdf

[9] Declercq ER, Sakala C, Corry MP, Applebaum S, Herrlich A. Major survey findings of Listening to Mothers^SM^ III: Pregnancy and Birth: Report of the third national U.S. survey of women's childbearing experiences. J Perinat Educ. 2014;23(1):9‐16. 10.1891/1058-1243.23.1.9 24453463 PMC3894594

[10] Bridges KM. Reproducing Race: An Ethnography of Pregnancy as a Site of Racialization. Berkeley, CA: University of California Press; 2011.

[11] Vedam S, Stoll K, Taiwo TK, et al. The Giving Voice to Mothers study: inequity and mistreatment during pregnancy and childbirth in the United States. Reprod Health. 2019;16(1):77. 10.1186/s12978-019-0729-2 31182118 PMC6558766

[12] Institute of Medicine Committee on Understanding and Eliminating Racial and Ethnic Disparities in Health Care ; Smedley BD, Stith AY, Nelson AR, eds. Unequal Treatment: Confronting Racial and Ethnic Disparities in Health Care. Washington, DC: National Academies Press; 2003. 25032386

[13] Slaughter‐Acey JC, Sealy‐Jefferson S, Helmkamp L, et al. Racism in the form of micro aggressions and the risk of preterm birth among black women. Ann Epidemiol. 2016;26(1):7‐13.e1. 10.1016/j.annepidem.2015.10.005 26549132 PMC4688115

[14] Duru OK, Harawa NT, Kermah D, Norris KC. Allostatic load burden and racial disparities in mortality. J Natl Med Assoc. 2012;104(1‐2):89‐95. 10.1016/s0027-9684(15)30120-6 22708252 PMC3417124

[15] Centers for Disease Control and Prevention . User Guide to the 2016 Period Linked Birth/Infant Death Public Use File. Hyattsville, MD: National Center for Health Statistics, Centers for Disease Control and Prevention; 2018. https://ftp.cdc.gov/pub/Health_Statistics/NCHS/Dataset_Documentation/DVS/periodlinked/LinkPE16Guide.pdf

[16] Hoyert DL, Miniño AM. Maternal mortality in the United States: changes in coding, publication, and data release, 2018. Natl Vital Stat Rep. 2020;69(2):1‐18. 32510319

[17] Petersen EE, Davis NL, Goodman D, et al. Racial/ethnic disparities in pregnancy‐related deaths ‐ United States, 2007‐2016. MMWR Morb Mortal Wkly Rep. 2019;68(35):762‐765. 10.15585/mmwr.mm6835a3 31487273 PMC6730892

[18] Corbin J, Strauss A. Basics of Qualitative Research: Techniques and Procedures for Developing Grounded Theory. 3rd ed. Los Angeles, CA: SAGE Publications; 2008.

[19] Wallerstein N, Duran B. Community‐based participatory research contributions to intervention research: the intersection of science and practice to improve health equity. Am J Public Health. 2010;100(suppl 1):S40‐S46. 10.2105/AJPH.2009.184036 20147663 PMC2837458

[20] Kruk ME, Gage AD, Arsenault C, et al. High‐quality health systems in the Sustainable Development Goals era: time for a revolution. Lancet Glob Health. 2018;6(11):e1196‐e1252. 10.1016/S2214-109X(18)30386-3 30196093 PMC7734391

[21] Jones CP. Levels of racism: a theoretic framework and a gardener's tale. Am J Public Health. 2000;90(8):1212‐1215. 10936998 10.2105/ajph.90.8.1212 PMC1446334

[22] powell aj, Menendian S. The problem of othering: towards inclusiveness and belonging. Othering & Belonging. 2016;1(1):14‐40.

[23] Gwatkin DR, Ergo A. Universal health coverage: friend or foe of health equity? Lancet. 2011;377(9784):2160–2161. 21084113 10.1016/S0140-6736(10)62058-2

[24] powell aj, Menendian S, Ake W. Targeted Universalism: Policy and Practice. Berkeley, CA: Haas Institute; 2019. https://belonging.berkeley.edu/targeteduniversalism

[25] Maxwell JA. Qualitative Research Design: An Interactive Approach. 3rd ed. Los Angeles, CA: SAGE Publications; 2013.

[26] Jacobsen SK, Bouchard GM, Emed J, Lepage K, Cook E. Experiences of “being known” by the healthcare team of young adult patients with cancer. Oncol Nurs Forum. 2015;42(3):250‐256. 10.1188/15.ONF.250-256 25901377

[27] Hildingsson I, Karlström A, Rubertsson C, Haines H. Women with fear of childbirth might benefit from having a known midwife during labour. Women Birth. 2019;32(1):58‐63. 10.1016/j.wombi.2018.04.014 29773474

[28] Attanasio LB, McPherson ME, Kozhimannil KB. Positive childbirth experiences in U.S. hospitals: a mixed methods analysis. Matern Child Health J. 2014;18(5):1280‐1290. 10.1007/s10995-013-1363-1 24072597 PMC3966989

[29] Hodnett ED. Pain and women's satisfaction with the experience of childbirth: a systematic review. Am J Obstet Gynecol. 2002;186(suppl 5):S160‐S172. 10.1067/mob.2002.121141 12011880

[30] Bowser DH, Hill K. Exploring Evidence for Disrespect and Abuse in Facility‐Based Childbirth. Report of a Landscape Analysis. USAID Translating Research into Action Project. Boston, MA: Harvard School of Public Health; 2010.

[31] Altman MR, Oseguera T, McLemore MR, Kantrowitz‐Gordon I, Franck LS, Lyndon A. Information and power: women of color's experiences interacting with health care providers in pregnancy and birth. Soc Sci Med. 2019;238:112491. 10.1016/j.socscimed.2019.112491 31434029

[32] Weber M. Bureaucracy. In: From Max Weber: Essays in Sociology. Oxford University Press; 1946:196‐244.

[33] Foucault M. Discipline and Punish: The Birth of the Prison. New York: Vintage Books; 1979.

[34] Scott KA, Britton L, McLemore MR. The ethics of perinatal care for Black women: dismantling the structural racism in “mother blame” narratives. J Perinat Neonatal Nurs. 2019;33(2):108‐115. 10.1097/JPN.0000000000000394 31021935

[35] Steele CM, Aronson J. Stereotype threat and the intellectual test performance of African Americans. J Pers Soc Psychol. 1995;69(5):797‐811. 10.1037/0022-3514.69.5.797 7473032

[36] Abdou CM, Fingerhut AW. Stereotype threat among Black and white women in health care settings. Cultur Divers Ethnic Minor Psychol. 2014;20(3):316‐323. 10.1037/a0036946 25045944 PMC5449200

[37] Alcorn KL, O'Donovan A, Patrick JC, Creedy D, Devilly GJ. A prospective longitudinal study of the prevalence of post‐traumatic stress disorder resulting from childbirth events. Psychol Med. 2010;40(11):1849‐1859. 10.1017/S0033291709992224 20059799

[38] Soet JE, Brack GA, DiIorio C. Prevalence and predictors of women's experience of psychological trauma during childbirth. Birth. 2003;30(1):36‐46. 10.1046/j.1523-536X.2003.00215.x 12581038

[39] Reed R, Sharman R, Inglis C. Women's descriptions of childbirth trauma relating to care provider actions and interactions. BMC Pregnancy Childbirth. 2017;17(1):21‐21. 10.1186/s12884-016-1197-0 28068932 PMC5223347

[40] McLemore MR, Altman MR, Cooper N, Williams S, Rand L, Franck L. Health care experiences of pregnant, birthing and postnatal women of color at risk for preterm birth. Soc Sci Med. 2018;201:127‐135. 10.1016/j.socscimed.2018.02.013 29494846

[41] Owens DC, Fett SM. Black maternal and infant health: historical legacies of slavery. Am J Public Health. 2019;109(10):1342‐1345. 10.2105/AJPH.2019.305243 31415204 PMC6727302

[42] Wilson E, Kenny A, Dickson‐Swift V. Ethical challenges of community based participatory research: exploring researchers’ experience. Int J Soc Res Method. 2018;21(1):7‐24. 10.1080/13645579.2017.1296714 29235941

[43] Altman MR, McLemore MR, Oseguera T, Lyndon A, Franck LS. Listening to women: recommendations from women of color to improve experiences in pregnancy and birth care. J Midwifery Womens Health. 2020;65(4):466‐473. 10.1111/jmwh.13102 32558179

